# Integrative bioinformatics screening of a M2 macrophage-associated gene signature for osimertinib resistance

**DOI:** 10.3389/fgene.2026.1928986

**Published:** 2026-09-28

**Authors:** Yajie Huang, Yaozhong Zhang, Jian Shi

**Affiliations:** 1 Department of Medical Oncology, The Fourth Hospital of Hebei Medical University, Shijiazhuang, China; 2 Department of Infectious Diseases, The Fourth Hospital of Hebei Medical University, Shijiazhuang, China

**Keywords:** lung adenocarcinoma, machine learning, macrophage, osimertinib resistance, RRBP1

## Abstract

**Introduction:**

Although the third-generation epidermal growth factor receptor tyrosine kinase inhibitor (EGFR-TKI) Osimertinib is widely used in lung adenocarcinoma (LUAD), acquired resistance to this agent remains a major clinical challenge perplexing the academic community. While the genomic drivers of osimertinib resistance (OR) have been relatively well characterized, the non-genetic contributions of the tumor microenvironment (TME), especially the role of macrophage-mediated remodeling remains incompletely understood.

**Methods:**

To address this gap, a total of 6 lung cancer tissue samples were collected from a cohort of 6 NSCLC patients and subjected to single-cell RNA sequencing (scRNA-seq). Weighted gene co-expression network analysis (WGCNA) was performed on Gene Expression Omnibus (GEO) data to screen out OR-associated gene modules. The intersection of scRNA-seq-derived differentially expressed genes (DEGs) and module genes was used to identify M2 macrophage- and OR-related DEGs (MORGs). An ensemble machine learning framework encompassing 112 algorithm combinations was applied to construct a gene signature. Survival analysis was conducted in the EGFR-mutant TCGA cohort. In vitro cell experiments were further performed for functional validation.

**Results:**

Single-cell RNA sequencing firstly revealed an increased proportion of M2 macrophages in the OR group, and M2 macrophage-specific DEGs were identified between OR and non-OR groups. A total of seven MORGs were obtained by intersecting DEGs and OR-related module genes. A 7-gene signature was successfully established via machine learning. Survival analysis demonstrated that RRBP1 showed the strongest association with overall survival and was selected for further investigation. In vitro experiments confirmed that RRBP1 was significantly upregulated in the H1975OR cells. Knockdown of RRBP1 reduced cell viability and migration, lowered the IC50 of osimertinib, and increased the expression of ER stress markers such as GRP78 and CHOP.

**Conclusion:**

In summary, our study identified a 7-MORGs signature for OR, among which RRBP1 may be a candidate regulator for OR.

## Introduction

Worldwide, lung cancer was the leading cause of cancer-related death and accounted for nearly half a million deaths per year and non-small cell lung cancer (NSCLC) constitutes about 85% of all cancers diagnosed. Lung adenocarcinoma (LUAD) represents 40%–50% of the NSCLC diagnosis accounting for 23.6% of all cancer-related deaths (Sung et al., 2021; Travis et al., 2015). The discovery of EGFR mutations is considered a turning point in treatment of advanced LUAD (Lynch et al., 2004). Treated with first-or second-generation EGFR tyrosine kinase inhibitors (TKIs) have markedly improved clinical outcomes compared with conventional chemotherapy (Mok et al., 2009), but their long-term efficacy is almost always undermined by the development of acquired resistance, with the secondary T790M mutation being the most common and clinically important resistance mechanism, occurring in 50%–60% of cases (Yu et al., 2013; Oxnard et al., 2011). Therefore, osimertinib, a third-generation EGFR-TKI specifically designed to target both sensitizing EGFR mutations and the T790M variant, represents a major advance. Osimertinib has firmly established itself as the definitive standard treatment (Cross et al., 2014; Janne et al., 2015), with clear, well-documented efficacy in untreated advanced EGFR mutant NSCLC (Soria et al., 2018; Ramalingam et al., 2020). However, it is also undeniable that osimertinib resistance (OR) is virtually inevitable, with disease progression generally occurring within 18–20 months of first-line therapy (Ramalingam et al., 2020; Oxnard et al., 2018), hence it represents a major unmet clinical need. Although cell-intrinsic mechanisms such as tertiary EGFR mutations and bypass pathway activation are well characterized (Leonetti et al., 2019), they do not satisfactorily account for the clinical heterogeneity of treatment failure, which logically leads to the compelling conclusion: a more detailed investigation of the tumor microenvironment (TME) is urgently needed.

The TME is now clearly established as a major determinant of drug resistance, and among its cellular components, tumor-associated macrophages (TAMs) play a central role in creating an immunosuppressive niche that favors tumor progression and reduces drug sensitivity (Cassetta and Pollard, 2018). More importantly, there is growing evidence that the dynamic reprogramming of macrophage phenotypes from tumor-suppressive to pro-tumorigenic contributes directly to the resistance of LUAD to Osimertinib (Kim et al., 2025). Thus, it must be acknowledged that the potential molecular crosstalk between OR malignant cells and macrophage remains to be fully elucidated.

The development of high-throughput transcriptomic technologies has provided unprecedented opportunities to study complex intercellular interactions, and thus it is natural to consider the strengths and limitations of bulk RNA sequencing (bulk RNA-seq), which gives a global view of the transcriptome in clinical cohorts but cannot reliably resolve cell-type-specific contributions (Finotello and Trajanoski, 2018). In sharp contrast, single-cell RNA sequencing (scRNA-seq) has emerged as a revolutionary tool for dissecting cellular heterogeneity and identifying resistance-associated cell states within the TME (Lambrechts et al., 2018). Therefore, integrating these two modalities offers a direct and powerful way to link cellular resolution with clinical relevance.

Because of the exponential growth of multi-omics datasets requires increasingly advanced analytical methods, machine learning (ML) algorithms have shown remarkable success in extracting high-dimensional genomic data into reliable, reproducible predictive signatures that help patient stratification (Li et al., 2022). In this research, we adopted a three-step sequential strategy including bioinformatic screening, clinical correlation, and *in vitro* experiment validation. First, we integrated single-cell and bulk RNA-sequencing data and identified 15 macrophage- and OR-related genes (MORGs). Then, we then applied a machine learning framework comprising 112 algorithm combinations to construct a 7 MORGs signature. Furthermore, we performed survival analysis to examine the correlation of these signature genes with patient survival in the EGFR-mutant TCGA cohort, which served to prioritize candidates with both diagnostic and prognostic relevance for downstream studies. Third, with RRBP1 emerging as the core candidate from both analyses, we performed *in vitro* validation to explore its role in OR. The study workflow is summarized in Figure 1.

**FIGURE 1:**
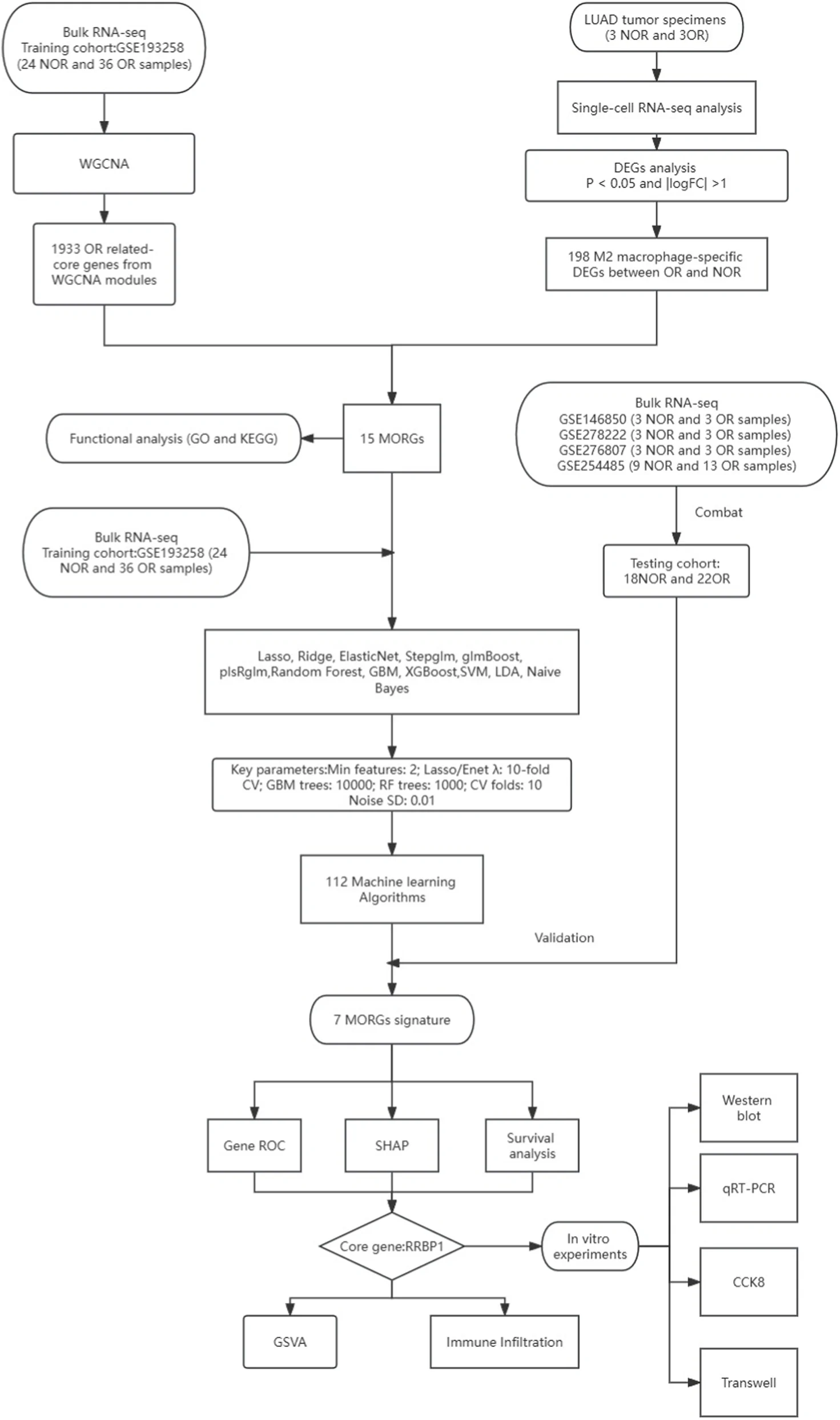
The workflow of the study.

## Materials and methods

### Clinical sample collection and processing

This study was approved by the Institutional Ethics Committee of The fourth hospital of Hebei Medical University (approval no.2026KS096), and written informed consent was obtained from all participants before sample collection. Tumor specimens were collected from six patients with pathologically confirmed LUAD who had received osimertinib treatment (Table SI) between January 2024 and August 2026. Patients were categorized into non-osimertinib-resistant (NOR) (n = 3) and osimertinib-resistant (OR) (n = 3) groups according to (Sung et al., 2021): Clinical and radiographic assessments based on Response Evaluation Criteria in Solid Tumors (RECIST), version 1.1 (Travis et al., 2015). Patients in the NOR group were from patients who achieved partial or complete response (according to RECIST 1.1 criteria) with progression-free survival (PFS) longer than 6 months after osimertinib treatment, the samples were collected before osimertinib treatment (Lynch et al., 2004). OR was defined as radiologically confirmed progressive disease according to RECIST version 1.1 after an initial objective response or durable clinical benefit from osimertinib. Tumor tissues were obtained by surgical resection or computed tomography-guided percutaneous biopsy. Specimens intended for single-cell RNA sequencing (scRNA-seq) were immediately placed in prechilled sterile transport medium, transported to the laboratory on ice, and processed promptly after collection. All samples were independently reviewed by experienced pathologists, and specimens with a tumor-cell proportion of at least 60% were included for subsequent analysis.

### Preparation of single-cell suspensions

Tissues were first rinsed in prechilled sterile PBS to remove surface blood clots and adipose tissue. Under aseptic conditions, tissues were minced into approximately 1 mm^3^ fragments and incubated in 5 mL RPMI-1640 medium supplemented with Dispase II (123456, Roche, Germany; 2 mg/mL) and DNase I (DN25, Sigma-Aldrich, USA; 0.1 mg/mL). The digestion mixture was incubated at 37 °C with agitation at 120 rpm for 30 min, with gentle pipetting every 10 min to facilitate cell dissociation. The resulting cell suspension was filtered through a 40 μm nylon mesh (CLS431750, Corning, USA), and cells were collected by centrifugation at 300 *g* for 5 min. The pellet was subsequently washed twice with PBS containing 0.04% BSA to reduce cell aggregation and mortality. Cell viability was assessed by staining with trypan blue (15250061, Thermo Fisher Scientific, USA) and counting with an automated cell counter (Countess II FL, Thermo Fisher Scientific, USA). Only cell suspensions with viability ≥85% and achieving single-cell dispersion were used for library construction.

### Single-cell RNA sequencing (scRNA-seq) library construction and sequencing

Qualified single-cell suspensions were diluted to approximately 1,000 cells/μL with a target capture of 8,000–10,000 cells per sample. Cells were loaded onto the 10x Genomics Chromium system (Chromium Controller, 10x Genomics, USA), and scRNA-seq libraries were constructed using the Chromium Next GEM Single Cell 3′ Reagent Kit v3.1 (1000128, 10x Genomics, USA) according to the manufacturer’s protocol. Library quality was assessed by measuring DNA concentration with a Qubit 4.0 fluorometer (Q33238, Thermo Fisher Scientific, USA) and determining fragment size distribution using an Agilent 2100 Bioanalyzer (G2939B, Agilent, USA), with a major peak at approximately 400 bp. Qualified libraries were sequenced on an Illumina NovaSeq 6000 platform (Illumina, USA) in paired-end 150 bp mode, with a sequencing depth of at least 50,000 reads per cell.

### ScRNA-seq analysis

Dimensionality reduction and clustering analyses were performed using the Seurat package (version 4.x) in R. Quality control was conducted by filtering cells with 200–4,000 detected genes and mitochondrial gene content <20%, followed by removal of genes expressed in fewer than 3 cells. Gene expression matrices were normalized using the log-normalization method, with variance stabilization performed on the 2,000 most highly variable genes. Next, the high-dimensional dataset was normalized, and key information was extracted using principal component analysis (PCA) to convert it into a low-dimensional representation, distributed on a two-dimensional plane. The Limma and sva packages were used for batch effect correction to eliminate potential batch effects. Based on the elbow plot, the first 31 principal components were retained for downstream analysis. Cell clustering was performed using the shared nearest neighbor (SNN) algorithm with Louvain optimization, and visualization was achieved through t-distributed stochastic neighbor embedding (t-SNE) in two-dimensional space. Differential expression analysis was performed using Seurat’s FindAllMarkers function with the Wilcoxon rank-sum test. Cluster-specific differentially expressed genes (DEGs) were identified with thresholds of adjusted P < 0.05 and |log_2_ fold change|>1. Cell type annotation was carried out by matching cluster-specific marker genes to canonical cell signatures from the CellMarker 2.0 database (http://117.50.127.228/CellMarker/index.html). We further performed differential expression analysis between the OR and NOR groups to identify key cell-type-specific marker genes associated with therapeutic response.

### Data acquisition and preprocessing

Five bulk RNA-seq datasets were downloaded from the Gene Expression Omnibus (GEO) database (https://www.ncbi.nlm.nih.gov/geo/) on 2 February 2026. GSE193258 (24 NOR and 36 OR samples) served as the training set. The four cohorts: GSE146850 (3 NOR and 3 OR samples), GSE278222 (3 NOR and 3 OR samples), GSE276807 (3 NOR and 3 OR samples), and GSE254485 (9 NOR and 13 OR samples) were designated as independent testing cohorts. Microarray data were normalized using robust multi-array average (RMA), and raw RNA-seq read counts were normalized to fragments per kilobase of exon per million mapped reads (FPKM). Batch effects were corrected using the ComBat function from the sva R package (version 3.58.0; https://bioconductor.org/packages/release/bioc/html/sva.html), with correction efficacy confirmed by Principal Component Analysis (PCA) (Zhang et al., 2020). Additionally, RNA-seq count data and corresponding clinical information for EGFR-mutant LUAD were obtained from The Cancer Genome Atlas (TCGA) database (https://portal.gdc.cancer.gov) on 2 February 2026. Data were converted to transcripts per million (TPM) and log_2_ (TPM+1) transformed. Samples lacking matched clinical and transcriptomic data were excluded, yielding a final TCGA dataset of 66 samples. As both TCGA and GEO provide fully de-identified public data, individual participant identification was not possible.

### WGCNA

The usage of WGCNA package (version no. 1.74; https://bioconductor.org/packages//2.7/bioc/html/limma.html) in R constructed a signed co-expression network, first selecting an appropriate soft-thresholding power with the pickSoftThreshold function to achieve a scale-free topology, then partitioning modules with dynamic tree cutting (minimum module size of 200 genes), and assigning unassigned genes to the “grey” module. To connect the clusters with OR status, Pearson correlations were calculated between each module eigengene and OR status. Finally, Gene Significance (GS) and Module Membership (MM) were computed to identify candidate genes related to OR.

### MORGs identification

To precisely identify the MORGs, this study utilized two gene sets (Sung et al., 2021): DEGs in M2 macrophages comparing OR vs. NOR samples (identified by scRNA-seq); and (Travis et al., 2015) genes from the module significantly associated with OR identified by WGCNA. The overlap was visualized using a Venn diagram generated with the “VennDiagram” package.

### Functional enrichment analysis

Gene Ontology (GO) and Kyoto Encyclopedia of Genes and Genomes (KEGG) pathway enrichment analyses were performed for the candidate MORGs using the “clusterProfiler” R package (version 4.14.6) (Yu et al., 2012). GO enrichment analysis included biological process (BP), cellular component (CC), and molecular function (MF) categories. Gene symbols were converted to Entrez gene identifiers using the “org.Hs.eg.db” annotation package (version3.20.0), and KEGG pathway enrichment analysis was conducted using the human gene set (organism = “has”). P values were adjusted for multiple testing using the Benjamini-Hochberg method. Terms with P value < 0.05 were considered enriched. The enriched terms were visualized using bubble plots and dot plots generated with the “ggplot2” R package (version 3.5.1).

### Machine learning-based MORGs signature screening

We adopted 15 candidate MORGs obtained from the intersection of differentially expressed genes and WGCNA module genes (described above) as the predefined feature set for subsequent diverse machine learning frameworks (Liu et al., 2022). A comprehensive panel of twelve distinct machine learning algorithms was implemented, spanning regularization methods (Lasso, Ridge, ElasticNet), generalized linear model approaches (Stepglm, glmBoost, plsRglm), ensemble learning techniques (Random Forest, GBM, XGBoost), pattern recognition models (SVM, LDA, Naive Bayes), and gradient boosting frameworks. A two-stage feature selection strategy was adopted. First, for each machine learning method capable of feature selection (e.g., Lasso, Ridge, Elastic Net, Stepwise GLM, Random Forest, GBM), the algorithm was run independently on the training set to automatically identify significant variables. The minimum number of variables was set to 2, if fewer variables were selected, they were supplemented to the threshold based on t-test P-values. All variable screening methods were uniformly implemented via the custom function RunML, with hyperparameters optimized using 10-fold cross-validation (e.g., Lasso/Enet/GBM). Core parameters, including the alpha of Elastic Net, the number of trees for GBM (n.trees = 10,000), and the number of cross-validation folds (Ramalingam et al., 2020), were set based on empirical evidence. Based on the aforementioned feature sets, each algorithm was used to construct its final model. Commonly used methods included Lasso regression (glmnet, alpha = 1), Ridge regression (glmnet, alpha = 0), Elastic Net (glmnet, alpha between 0 and 1), Random Forest (randomForestSRC, ntree = 1,000, nodesize = 5), GBM (gbm, n.trees = 10,000, interaction. Depth = 3, shrinkage = 0.001, cv.folds = 10), Support Vector Machine (e1071), Naïve Bayes (e1071), Partial Least Squares Generalized Linear Model (plsRglm), and BART. For combined approaches (e.g., “Lasso + SVM”, “glmBoost + Ridge”), the former method was first used for feature selection, followed by model construction using the latter. Certain methods (e.g., stepwise regression) employed stepwise selection (forward, backward, or bidirectional, with the direction parameter set to “forward”, “backward”, or “both”). Key Parameters and Thresholds were set as (Sung et al., 2021): Minimum number of features: 2 (Travis et al., 2015). Lasso/Enet regularization parameter: determined automatically via 10-fold cross-validation (Lynch et al., 2004). Number of GBM trees: 10,000 (Mok et al., 2009). Number of Random Forest trees: 1,000 (Yu et al., 2013). Number of cross-validation folds: 10 (Oxnard et al., 2011). Noise standard deviation: 0.01. After model training, the testing set was used for external validation. Models with high average area under the receiver operating characteristic curve (AUC) and a moderate number of included genes were prioritized to balance predictive accuracy and clinical interpretability. AUC heatmaps generated with the ComplexHeatmap package were used to visualize the cross-cohort diagnostic stability of each model. The model with the highest AUC was selected as the optimal classifier for deriving the final MORGs signature, and then naturally proceeds to address the model’s “black-box” nature by integrating a SHapley Additive exPlanations (SHAP) framework to quantify feature contributions and thus resolve issues of model opacity. To decode the decision-making process of the stacking ensemble, a game-theoretic SHAP approach is elegantly employed: it assigns each feature an SHAP value that exactly represents its marginal effect, making feature importance quantification both rigorous and interpretable.

### Expression and prognostic value and functional enrichment analysis of MORGs signature genes

Boxplots were applied to compare the expression levels of the MORGs signature between the NOR and OR groups. For survival analysis within the TCGA cohort, patients were stratified into high- and low-risk subgroups according to the median expression of ML-derived biomarkers. Survival differences were visualized by Kaplan–Meier curves, and inter-group comparisons were performed using the log-rank test. Univariate Cox regression analysis was utilized to calculate hazard ratios (HR) alongside their corresponding 95% confidence intervals (CI). A P-value less than 0.05 was regarded as the threshold for statistical significance. GSVA enrichment analyses were performed using the “GSVA” packages (Hänzelmann et al., 2013), with reference gene sets obtained from the Molecular Signatures Database (MSigDB; http://www.gsea-msigdb.org/gsea/msigdb), including “c2.cp.kegg.v7.4.symbols.gmt” for KEGG pathways and “c5.go.v7.4.symbols.gmt” for GO functional annotations. These analyses were conducted to systematically explore the enrichment of biological pathways and functional modules associated with the expression levels of core genes.

### Immune cell infiltration and immune correlation of the core gene

The xCell deconvolution algorithm was employed to estimate the infiltration abundance of 64 immune and stromal cell subtypes from transcriptomic data (Aran et al., 2017), and the cellular composition of the tumor immune microenvironment was compared between the OR and NOR groups. In addition, Spearman correlation analysis was conducted to characterize the correlation between core gene expression and the infiltration scores of pivotal immune cell subsets.

### Cell culture and establishment of osimertinib resistance

The NCI-H1975 cell line (cat. No. CC0206; https://www.cellcook.com/product/show8121.html) was obtained from Cellcook Biotech (Guangzhou, China). Cell line authentication was performed by the supplier using short tandem repeat (STR) profiling to exclude cross-contamination and misidentification. Cells were cultured in RPMI-1640 supplemented with 10% fetal bovine serum (FBS; Gibco; Thermo Fisher Scientific, Inc.) at 37 °C in a 5% CO_2_ incubator (SANYO Electric Co., Ltd.). Osimertinib-resistant cells (H1975OR) were established by exposing parental cells to incrementally increasing concentrations of osimertinib (0.35 μM–5 μM) over a 6-month period (Tian et al., 2022). Resistance was maintained in drug-free medium prior to experiments. The Resistance Index (RI) was defined as the IC_50_ ratio between resistant and parental cell lines.

### siRNA transfection

Small interfering RNAs (siRNAs) targeting RRBP1(si-RRBP1) and the corresponding non-targeting control (si-NC) were obtained from NewHelix Co., Ltd., and their sequences are given in Table SII. Cells were seeded in 6-well plates at 5 × 10^5^ cells/well and grown in complete growth medium for 24 h until they reached 70%–80% confluence at the time of transfection. Transient transfection was then carried out with Lipofectamine RNAiMAX (Invitrogen; Thermo Fisher Scientific, Inc.). The procedure was carried out strictly according to the manufacturer’s protocol: siRNA (final concentration 50 nM) and the reagent were first pre-diluted in Opti-MEM (Gibco; Thermo Fisher Scientific, Inc.), mixed in a 1:1 ratio, and incubated for 15 min to allow proper siRNA-lipid complex formation. The complexes were then added dropwise to antibiotic-free medium, and after 6–8 h, the cells were refed with fresh complete growth medium. Knockdown efficiency was assessed by RT-qPCR 48 h post-transfection, with each experiment performed in triplicate to ensure reliable, reproducible results.

### Quantitative real-time PCR (RT-qPCR)

Total RNA was isolated using TRIzol reagent (Invitrogen; Thermo Fisher Scientific, Inc.), then cDNA was synthesized using FastKing gDNA Dispelling RT SuperMix (catalog no. KR118-02; Tiangen biotech Beijing Co., Ltd.) according to the manufacturer’s instructions. The reverse transcription procedure was: 42 °C for 15 min and then 3 min at 95 °C. RT was conducted by Beyofast SYBR™ Green qPCR mix (Beyotime Biotechnology) on a CFX96 Touch Real-Time PCR Detection System (Bio-Rad Laboratories, Inc.). The RT-qPCR system contained 10 μL 2×SYBR Green qPCR Master Mix, 0.6 μL forward primer, 0.65 μL reverse primer, 1 μL cDNA product, and 7.8 μL water nuclease-free. The reaction protocol was 95 °C for 30 s, followed by 40 cycles at 95 °C for 15 s and 60 °C for 30 s. The primers were synthesized by Servicebio (Wuhan, China). Target gene expression was normalized to the endogenous control GAPDH, and quantification was performed using the 2^–ΔΔCq^ method (Livak and Schmittgen, 2001). Primer sequences used in our experiments were as follows: *RRBP1* (F: 5′-CCC​AAG​GAG​GGC​AGA​AAT​ACA-3′, R: 5′-CCA​GAC​TTC​TTC​TTG​GCA​GG-3′); *GAPDH* (F: 5′-GGA​GCG​AGA​TCC​CTC​CAA​AAT-3′, R: 5′-GGC​TGT​TGT​CAT​ACT​TCT​CAT​GG-3′).

### Western blot analysis

Total protein was extracted using RIPA lysis buffer containing protease and phosphatase inhibitors (Beyotime, P0013B) supplemented with protease inhibitor cocktail (Roche, 04693159001) and phosphatase inhibitor cocktail (Roche, 04906837001). Protein concentration was determined using a BCA Protein Assay Kit (Thermo Fisher, 23227), and 30 μg of total protein per lane was loaded for SDS–PAGE separation, followed by transfer to PVDF membranes (Millipore, IPVH00010). Membranes were blocked with 5% nonfat milk (BD Difco, 232100) for 1 h at room temperature and incubated overnight with the following primary antibodies: RRBP1 (Cohesionbio, CQA2682, rabbit pAb, 1:1,000), CHOP (Cohesionbio, CMA5011, rabbit mAb, 1:1,000), GRP78 (Cohesionbio, CMA5244, rabbit mAb, 1:1,000), and GAPDH (Cohesionbio, CPA1460, rabbit mAb, 1:1,000). Membranes were then incubated with HRP-conjugated secondary antibodies for 1 h at room temperature: HRP–Goat Anti-Rabbit IgG (ABclonal, AS014, 1:5,000). Protein bands were visualized using ECL chemiluminescent substrate (Thermo, 34095), and densitometric analysis was performed using ImageJ, with GAPDH used for normalization. Each experiment included three biological replicates (n = 3).

### CCK-8 cell viability assay

Cell viability was assessed using the Cell Counting Kit-8 (CCK-8; Beyotime Biotechnology). Cells were seeded in 96-well plates (3,000 cells/well) and treated with osimertinib (1 nM–20 μM) for 48 h. CCK-8 reagent (10 µL) was added, and absorbance at 450 nm was measured after 2 h using a microplate reader (BioTek, USA). Each experiment was independently repeated at least three times (n ≥ 3), and data were expressed as mean ± standard deviation (SD). IC_50_ values were derived from dose-response curves using a four-parameter logistic regression model: $Y=\text{Bottom}+\frac{\text{Top}‐\text{Bottom}}{1+10^{\left(\text{LogIC}50‐X\right)\times \text{HillSlope}}}$ , with curve fitting.

### Cell migration assay

To evaluate migratory potential, we utilized Corning Transwell chambers (8.0-µm pore size). Cells were subjected to serum deprivation for 24 h prior to being seeded (1 × 10^5^ cells/well) in the upper compartment with serum-free medium. The lower compartment was filled with 600 µL of 10% FBS-containing medium to serve as the chemoattractant. Post-incubation (24 h), cells remaining on the upper membrane were removed via cotton swabs, while migrated cells were fixed with 4% paraformaldehyde and stained with 0.1% crystal violet. We captured images and counted cells in five randomly selected fields across three independent experiments.

### Statistical analysis

All statistical computations were executed in R (v4.3.0) and GraphPad Prism (v10.0). Unless otherwise noted, quantitative data are expressed as mean ± SD. For comparisons between two groups, independent-sample t-tests were applied to normally distributed data, whereas Mann–Whitney U tests were used for nonparametric data. For comparisons among multiple groups, one-way ANOVA followed by Tukey’s *post hoc* test was conducted; when normality assumptions were not met, the Kruskal–Wallis test with Dunn’s correction was employed. The value of P < 0.05 was considered statistically significant.

## Results

### Analysis at the single-cell level of LUAD

After quality control, 28,765 high-quality cells were retained for subsequent analysis, comprising 15,789 cells from NOR samples and 12,976 cells from OR samples (Figure 2A). Post-log-normalization and dimensionality reduction, cells were segregated into 22 transcriptionally distinct subpopulations (Figure 2B). Based on canonical marker gene expression patterns (Supplementary Figure S1), these clusters were annotated as follows: AT2 cells (clusters 0, 6, 11, 18), Secretory cells (clusters 1, 3, 5, 19, 21), AT1 cells (cluster 2), M2 Macrophages (clusters 4, 14), cDC2 (cluster 7), Chondrocytes (cluster 8), Stem-like/Persister cells (cluster 9), T cells (cluster 10), Ciliated cells (cluster 12), B cells (cluster 13), Endothelial cells (clusters 15, 20), Neuroendocrine-like Malignant cells (cluster 16), and Smooth Muscle cells (cluster 17) (Figure 2C). The proportional distribution of each cell type between the NOR and OR group is displayed in Figure 2D, which demonstrated an increase in the proportion of M2 macrophages in OR group compared with NOR group. Given the limited number of samples (OR = 3 and NOR = 3), the findings from the scRNA-seq analysis should be regarded as preliminary and warrant further validation in larger cohorts.

**FIGURE 2:**
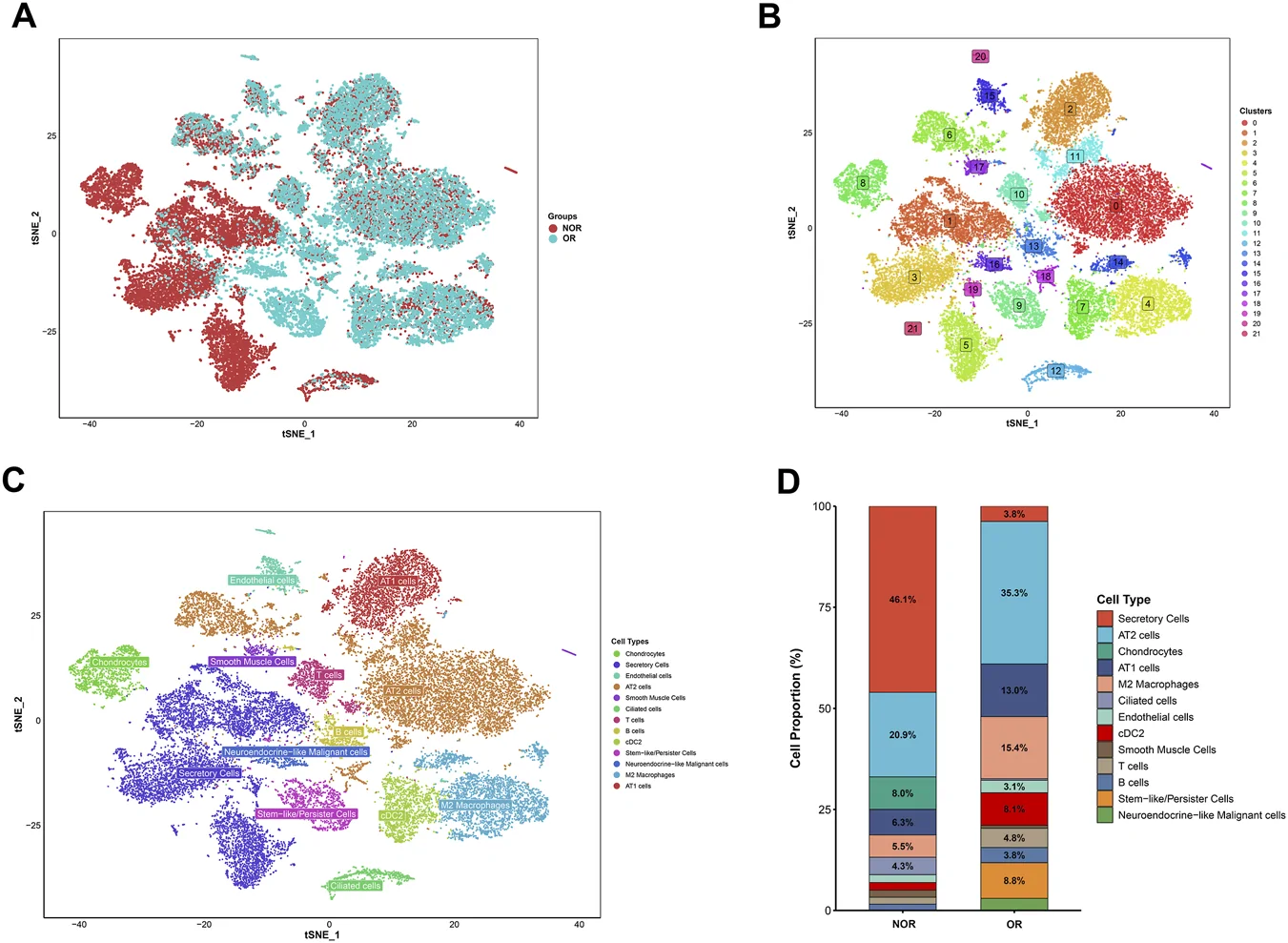
Cell type annotation based on distinct cellular markers and cellchat analysis. **(A)** T-distributed stochastic neighbor embedding (T-SNE) plot showing cells from the osimertinib-resistant and osimertinib-non-resistant groups. **(B)** TSNE visualization colored by 22 unsupervised clusters. **(C)** TSNE visualization annotated with 13 major cell types. **(D)** Proportional abundance of each cell type in the OR and NOR groups.

### Identification of MORGs and functional analysis

Differential expression analysis of the M2 macrophage subtype between NOR and OR group revealed 198 significant DEGs, visualized through volcano plots (Figure 3A). For WGCNA based on the GEO data, optimal soft thresholding power (β = 19) yielded 11 co-expression modules (Figures 3B,C). Among them, the MEturquoise module exhibited a negative correlation with the OR trait (r = −0.72, P = 9 × 10^−11^, Figure 3C). Further integration of differentially expressed genes with WGCNA module genes resulted in the identification of 15 candidate MORGs for downstream analysis (Figure 3D).

**FIGURE 3:**
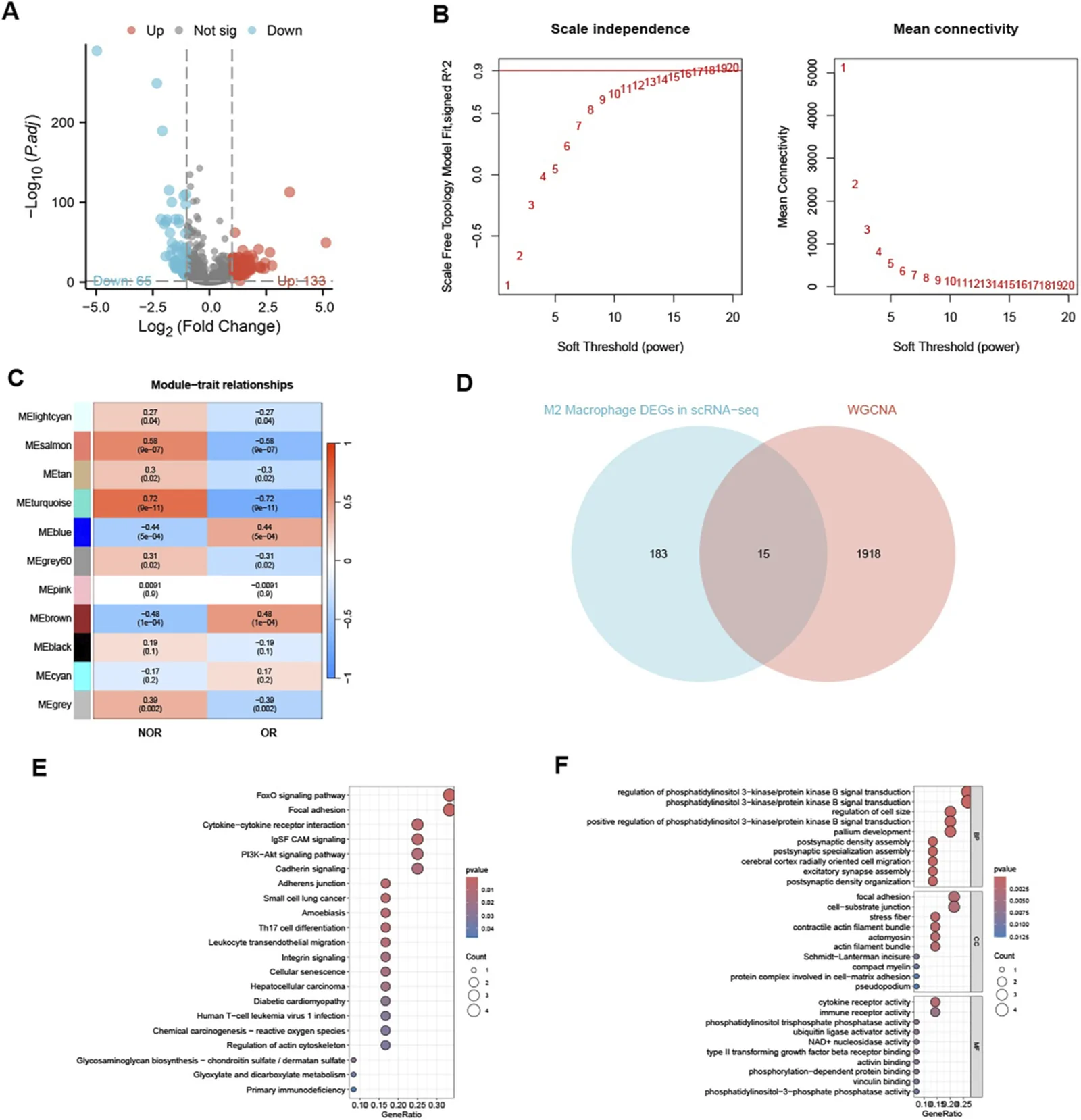
Identification and functional annotation of MORGs **(A)** Volcano plot of differentially expressed genes (DEGs). **(B)** WGCNA scale-free topology fit analysis identified a soft-thresholding power β = 19 as the minimum threshold required to construct an approximate scale-free network (R^2^ ≈ 0.9). **(C)** Correlation matrix depicting relationships among the 11 modules. **(D)** Venn diagram showed the overlap of genes identified through WGCNA and DEG analysis. **(E)** KEGG and Reactome pathway enrichment analysis. **(F)** GO functional enrichment analysis of MORGs, including biological process, cellular component, and molecular function categories.

To further investigate the biological characteristics of these 15 MORGs, GO and KEGG analyses were conducted. The top enriched KEGG pathways encompass FoxO signaling pathway, Focal adhesion, and Cytokine−cytokine receptor interaction (Figure 3E). The primary BPs identified include regulation of PI3K/Akt signal transduction, postsynaptic density assembly, and regulation of cell size. The CC terminology highlights stress fiber, contractile actin filament bundle, and actomyosin. The most prevalent MF include cytokine receptor and immune receptor activity (Figure 3F).

### Machine learning screening of MORGs signature

After batch correction using the ComBat algorithm, PCA analysis demonstrated improved consistency across datasets within the testing cohorts (Supplementary Figure S2). To refine feature selection from intersecting genes and enhance the predictive accuracy of the 15 MORGs, we established a comprehensive machine learning framework. A total of 112 predictive models were trained and validated using datasets derived from these genes, and the model with the highest AUC was selected for downstream analysis. Figure 4A presents a heatmap comparing the AUC values of different models across cohorts. Among these, RF (7 genes) model merged as the top-ranked position based on average AUC (Figure 4A). RF model identified 7 core genes: ACTN1, CSGALNACT2, RRBP1, TGFBR1, PTEN, APH1B and LAMB1. ROC curve analysis further validated the diagnostic value of these 7 genes for OR in train and test sets, respectively (Figures 4B,C). Among the 7 genes, RRBP1 exhibited the best single-gene performance (AUC = 0.812) in the testing cohort. SHAP interpretability assessment calculated the contribution of each gene to the predicted outcome and visualized the result (Figure 4D). The SHAP waterfall plot illustrates the influence of different features on the prediction model (Figure 4E). Among these, ACTN1 (SHAP = 0.285), PTEN (SHAP = 0.069) and RRBP1(SHAP = 0.052) was the top-three most influential predictor. Additionally, RRBP1 (78.5, Δ = −0.0945) and PTEN (15.9, Δ = −0.0584) functioned as exerted key negative regulatory effects, while ACTN1 (257, Δ = +0.219) and TGFBR1(22, Δ = +0.142) functioned as key positive regulators, impacting prediction values (f(x) falling below threshold expectation E [f(x)] = 0.963).

**FIGURE 4:**
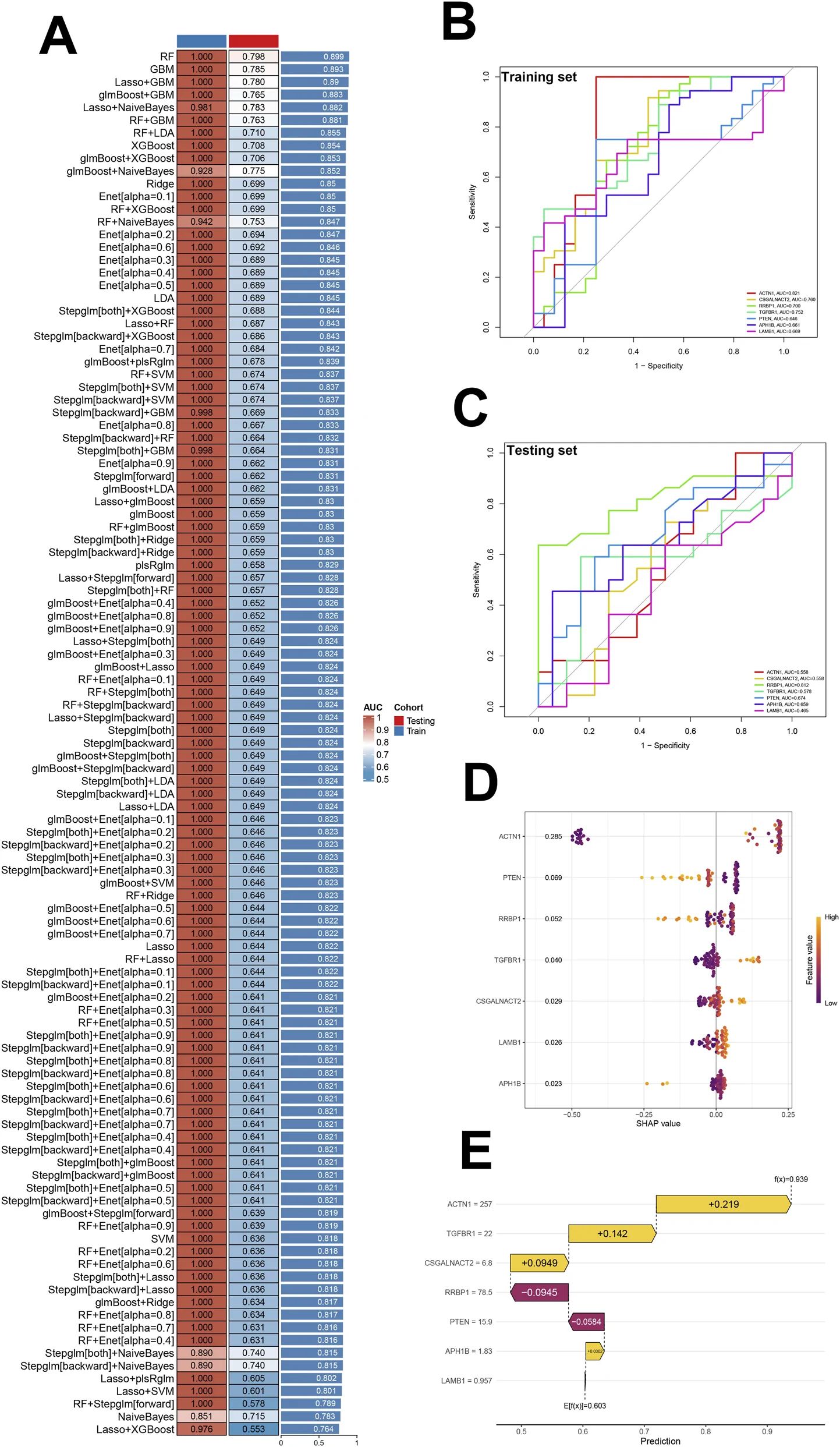
Machine learning based MORGs signature and SHAP validation. **(A)** All prediction models were calculated through a LOOCV framework, and the C-index was further calculated for each model on the training and validation datasets. **(B,C)** ROC curves of MORGs signature genes in train and testing sets. AUC indicates predictive performance. **(D)** SHAP summary dot plot. Each line represents a feature, and the x-axis represents SHAP values, indicating the impact of the feature on the outcome. Each dot represents an individual patient. The redder the color, the larger the feature value; the bluer the color, the smaller the feature value. **(E)** Waterfall plots show SHAP values for MORGs signature genes, indicating their impact on predictions.

### Expression patterns and prognostic value of MORGs signature

Boxplot analyses demonstrated that CSGALNACT2, RRBP1, ACTN1, and TGFBR1 were significantly upregulated in the OR group compared with the NOR group in the training cohort (P < 0.01; Figure 5A). In the testing cohort, however, only RRBP1 exhibited significantly higher expression in the OR group (P < 0.001; Figure 5B). Subsequently, univariate Cox proportional hazards regression was performed, and the results are summarized in the forest plot (Figure 6C). For each gene, patients were stratified into high- and low-expression subgroups using the median expression value as the cutoff. The hazard ratios (HR) for overall survival (OS), along with their 95% confidence intervals (CI) and corresponding P-values, are presented. Of the seven signature genes, only RRBP1 (P = 0.0171, HR = 2.79) and ACTN1 (P = 0.0336, HR = 2.44) reached statistical significance (Figure 5C). Kaplan–Meier survival analysis further confirmed that high expression of either RRBP1 or ACTN1 was significantly associated with poorer OS in EGFR-mutant LUAD patients (log-rank P = 0.0171 and 0.0336, respectively; Figures 5D,E). RRBP1 emerged as the only gene that was consistently upregulated in the OR group across both training and testing sets and simultaneously displayed the strongest prognostic association (highest HR, lowest *P*-value) among all signature genes, thereby warranting its selection as the key gene for subsequent analysis.

**FIGURE 5:**
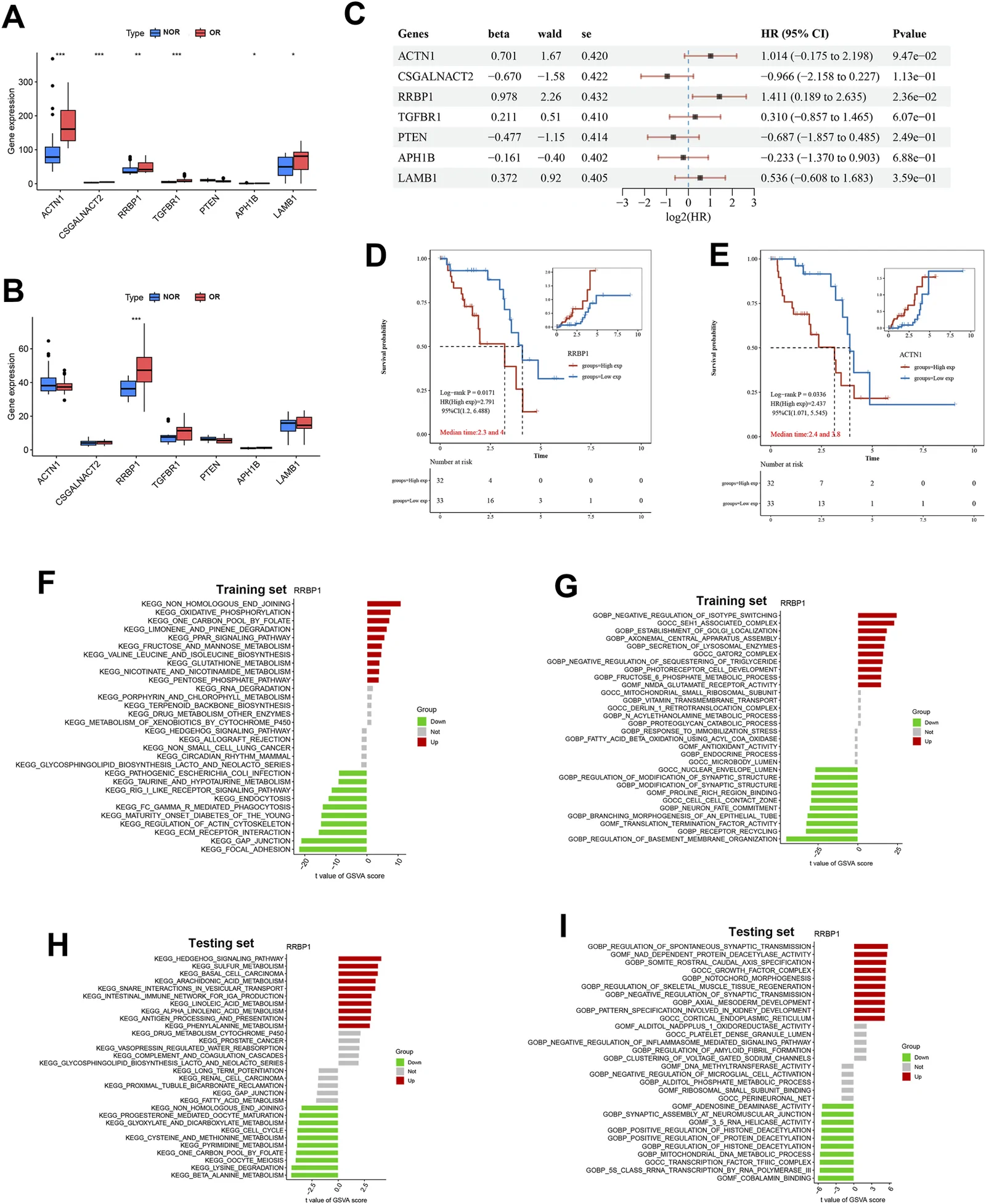
Expression patterns, prognostic value and functional analysis of MORGs signature genes. **(A)** Boxplots comparing expression between NOR and OR samples in the training set. **(B)** Validation of expression trends in the testing set. Significance levels: *P < 0.05, **P < 0.01, ***P < 0.001. **(C)** Univariate Cox analyses provide the p-values, HR, and CI for gene expression. **(D)** The KM survival curve of RRBP1 in TCGA data. **(E)** The KM survival curve of ACTN1 in TCGA data. **(F–I)** GSVA enrichment bar plots. **(F)** KEGG pathway enrichment of RRBP1 in the training cohort; **(G)** Gene Ontology (GO) enrichment of RRBP1 in the training cohort; **(H)** KEGG pathway enrichment of RRBP1 in the testing cohort; **(I)** GO enrichment of RRBP1 in the testing cohort.

**FIGURE 6:**
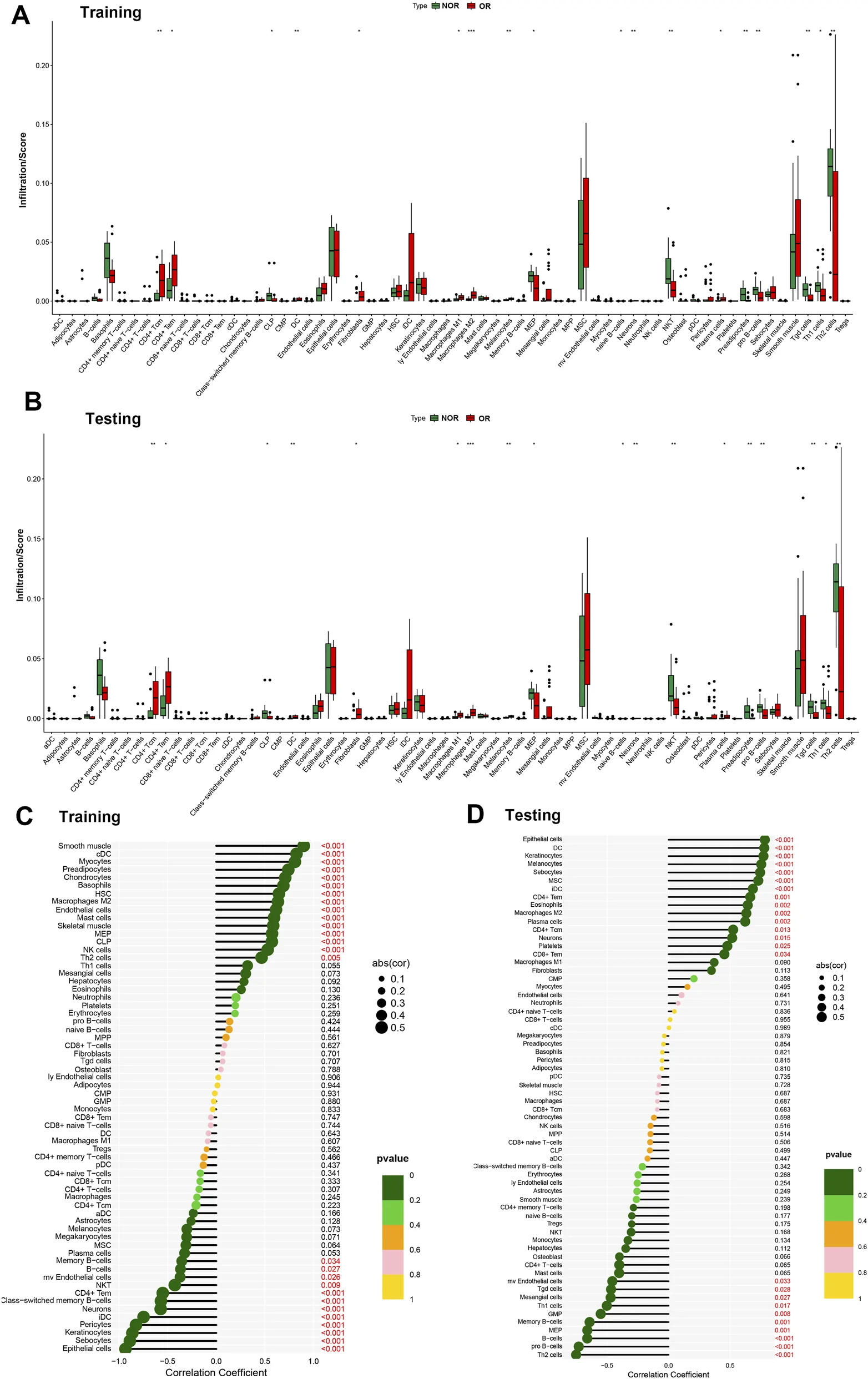
Immune cell infiltration features of the OR group and association of *RRBP1* expression with immune cell infiltration evaluated by the xCell algorithm **(A)** Box plots comparing xCell-derived immune cell infiltration levels between the NOR and OR groups in the training cohort. **(B)** Box plots comparing xCell-derived immune cell infiltration levels between the NOR and OR groups in the testing cohort. Significance levels: *P* < 0.05, P < 0.01, *P < 0.001. **(C)** Lollipop plot illustrating the correlation between *RRBP1* expression and infiltrating immune cell levels in the training cohort. **(D)** Lollipop plot illustrating the correlation between *RRBP1* expression and infiltrating immune cell levels in the testing cohort.

Single-gene gene set variation analysis (GSVA) was performed to explore the functional implications of RRBP1. In the training cohort, the KEGG analysis showed that high RRBP1 expression was associated with elevated activity of non-homologous end joining and oxidative phosphorylation, whereas focal adhesion and gap junction pathways were suppressed (Figure 5F). As for GO analysis, high RRBP1 expression was enriched in negative regulation of isotype switching, SEH1-associated complex, and establishment of Golgi localization, while regulation of basement membrane organization and translation termination factor activity were downregulated (Figure 5G). In the testing cohort, high RRBP1 expression was linked to increased activity of the Hedgehog signaling pathway and sulfur metabolism, whereas beta-alanine metabolism and lysine degradation were decreased (Figure 5H). GO analysis further revealed enrichment of regulation of spontaneous synaptic transmission, NAD-dependent protein deacetylase activity, and cortical endoplasmic reticulum (Figure 5I). Notably, the enrichment of cortical endoplasmic reticulum (ER) in the testing cohort, together with the established role of RRBP1 as an ER-associated ribosome-binding protein, prompted us to further investigate the potential involvement of RRBP1 in ER stress.

### RRBP1 expression is positively correlated with M2 macrophage infiltration

Correlation analysis demonstrated that OR status was significantly associated with multiple immune cell populations, particularly M2 macrophages were significantly upregulated in the OR group in both train and testing cohorts (P < 0.001, Figures 6A,B). In contrast, NKT cells with innate antitumor functions were significantly downregulated in the OR group in both cohorts (Figures 6A,B). Spearman analysis verified a positive correlation between RRBP1 expression and M2 macrophage infiltration scores across both cohorts (Figures 6C,D), indicating RRBP1 may shape an immunosuppressive microenvironment via promoting M2 macrophage infiltration. A significant negative correlation between RRBP1 level and NKT was shown in the training cohort (Figure 6C), with a consistent trend in the testing cohort (Figure 6D).

### RRBP1 knockdown reduces cell viability and migration in OR

To investigate the mechanisms underlying acquired resistance to EGFR-TKIs, we established an OR cell line (H1975OR) by subjecting parental H1975 cells to escalating concentrations of osimertinib over a 6-month period. Subsequent CCK-8 assays validated the resistant phenotype, with H1975OR cells exhibiting a profoundly increased tolerance to osimertinib compared to their parental counterparts (Figure 7A). Subsequent analyses involving protein quantification and qRT-PCR demonstrated that RRBP1 expression was increased in H1975OR cells (Figures 7B,C, P < 0.01), suggesting a potential role for RRBP1 in mediating drug resistance.

**FIGURE 7:**
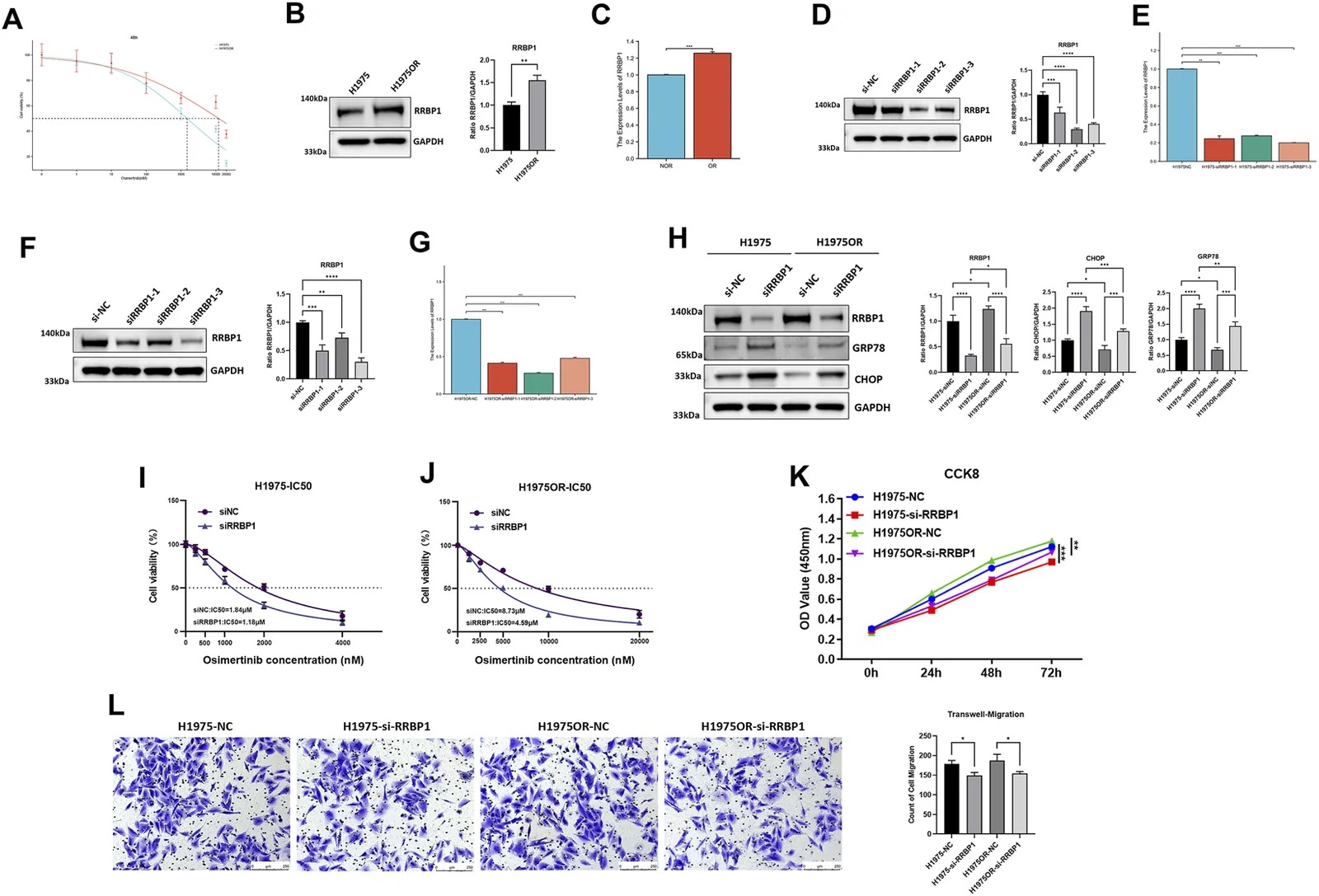
RRBP1 impact cell viability and migration. **(A)** Cell viability assessment using the CCK-8 assay to validate H1975OR construction. **(B)** RT-qPCR analysis of RRBP1 expression levels in H1975 and H1975-OR cells (n = 3). **(C)** RT-qPCR analysis of RRBP1 expression levels in H1975 and H1975-OR cells (n = 3). **(D)** The knockdown efficiency of RRBP1 in H1975 cells transfected with siRNA1/2/3 was detected using Western blot. **(E)** H1975 cells were transfected with RRBP1-siRNA or siNC, and the expression of RRBP1 was measured by RT-qPCR (n = 3). **(F)** The knockdown efficiency of RRBP1 in H1975OR cells transfected with siRNA1/2/3 was detected using Western blot. **(G)** H1975OR cells were transfected with RRBP1-siRNA or siNC, and the expression of RRBP1 was measured by RT-qPCR (n = 3). **(H)** Western blot analysis of GRP78 and CHOP protein expression in si-NC and si-RRBP1-KD in H1975 and H1975OR cells. **(I-J)** The IC50 of H1975 and H1975OR cells were detected by CCK-8 assay after RRBP1 knockdown,respectively. **(K)** CCK-8 assay showing that RRBP1 knockdown inhibits cell viability of H1975 or H1975OR cells. **(L)** Transwell migration assays showing reduced cell migration upon RRBP1 suppression. *P < 0.05, **P < 0.01, ***P < 0.001.

 To functionally validate whether RRBP1 contributes to the malignant progression predicted by our bioinformatic analysis, we performed loss-of-function assays using siRNA-mediated knockdown in both H1975 and H1975OR cells, and its knockdown efffciency was veriffed by Western blot and qPCR (Figures 7D–G). Selected siRNA3 was subjected to the subsequent functional experiments. Western blot analysis showed that GRP78 and CHOP protein levels of the siRRBP1 group were higher than the si-NC group in the H1975 and H1975OR cell (both P < 0.001, Figure7H), which suggested that RRBP1 downregulation may promote ER stress. The CCK-8 dose–response curves showed that the IC50 values of the H1975-siNC, H1975-siRRBP1, H1975OR-siNC and H1975OR-siRRBP1 groups were 1.84μM, 1.18μM, 8.73 μM and 4.59 μM, respectively (Figures 7I,J). In H1975 and H1975OR, RRBP1 knockdown decreased the IC50 compared with the NC group. RRBP1 knockdown signiffcantly suppressed cell viability of H1975 (P < 0.001) and H1975OR (P < 0.01), as examined by CCK-8 assays (Figure 7K). Furthermore, transwell assays demonstrated that RRBP1 knockdown markedly impaired the migratory capacity of these cells (Figure 7L, P < 0.05). 

## Discussion

OR is a major clinical bottleneck in the treatment of EGFR-mutant LUAD, and although genomic aberrations such as C797S mutations or MET amplification account for almost half of resistance cases (Passaro et al., 2021), the non-genetic mechanisms underpinning resistance, which involve TME remodeling, remain poorly understood (Han et al., 2024). Therefore, elucidating the underlying mechanism is of critical importance. Only few evidence indicates that macrophage play an important role in target therapy (Smith et al., 2014). A Recent study have reported an increased abundance of M2-like macrophages in TKI-resistant tissues, suggesting that an immunosuppressive microenvironment may develop upon the occur of TKI resistance (Fang et al., 2026). Furthermore, an autochthonous mouse model of lung cancer established by Jia et al. showed that EGFR-TKI treatment initially improved antitumor immunity, including increased CTLs, depleted Tregs, and inhibited M2 polarization, but these effects were transient. As treatment continued, these effects became gradually less stong, and immunosuppressive features such as accumulation of MDSCs gradually took over (Jia et al., 2019). However, our scRNA-seq and immune infiltration analysis only suggested that M2 macrophage was increased in the OR group compared with NOR group. Thus, further studies using humanized patient-derived xenograft models or sequential biopsies are needed to characterize how the tumor microenvironment evolves during EGFR-TKI treatment in human tissues.

Bulk RNA-seq is widely applied in previous research to explore the underlying mechanisms, which inherently masks cellular heterogeneity in the OR ecosystem (Maynard et al., 2020). In the study, we integrated scRNA-seq-derived DEGs with WGCNA-defined OR-associated modules from GEO data, then we identified a 7-gene signature using an ensemble of 112 algorithm combinations. RRBP1 showed the strongest association with overall survival in the EGFR-mutant TCGA cohort. Further experiments showed that RRBP1 was upregulated in osimertinib-resistant H1975 cells, and its knockdown attenuated viability and migration. The results were consistent with previous research which also reported RRBP1 closely correlates with cancer progression and poor prognosis (Telikicherla et al., 2012). Besides, RRBP1 is also known as an ER membrane protein involved in ribosome binding and translocation of nascent proteins, and has also been linked to ER stress and the unfolded protein response (UPR) (Wang et al., 2022). Our GSVA analysis revealed that high RRBP1 expression were enriched in the cortical ER, furthermore, RRBP1 knockdown enhanced ER stress markers in the Western blot result, the similar results were also reported that RRBP1 overexpression alleviates ER stress via enhancing GRP78 expression (Tsai et al., 2013), so its upregulation may be a adaptive response to the ER stress related to osimertinib. Notably, our finding complements the previous result of Fu et al., which also found RRBP1 as a new potentially pathogenic mutation of East-Asian Young Never-Smokers with LUAD (Fu et al., 2022). Our immune cell infiltration analysis indicated that RRBP1 expression was positively associated with M2 macrophage infiltration scores in both the training and testing cohorts, whereas a negative association with NKT cell abundance was observed. These results suggest that RRBP1 may contribute to an immunosuppressive tumor microenvironment, possibly through promoting M2 macrophage infiltration and reducing innate antitumor immune cells. Consistent with the possibility that RRBP1 is involved in macrophage-state regulation, a previous study showed that RRBP1 inhibition reduced the M1-like marker iNOS and the inflammatory cytokines TNF-α and IL-1β, while increasing the M2-like marker ARG1 and the pERK/ERK ratio in BV-2 and HMC3 microglial cells (Li et al., 2025). All above evidence indicated that deregulation of RRBP1 may contribute to OR.

Several limitations of this study should be acknowledged. First, the crosstalk between RRBP1 expression and macrophage polarization was not experimentally verified. Future studies using tumor-macrophage co-culture systems are needed to clarify how RRBP1 regulates macrophage polarization. Second, our scRNA-seq cohort was small (n = 6). Nevertheless, this dataset provided high-resolution insights into the single-cell immune landscape and was used primarily as a discovery resource. Third, immune infiltration was estimated using the xCell algorithm, which provides *in silico* predictions rather than direct measurements of immune cell abundance, so these results should be interpreted with caution. Accordingly, the proposed link between RRBP1 and an immunosuppressive tumor microenvironment remains preliminary and warrants direct experimental validation, such as immunohistochemistry, flow cytometry, or co-culture assays. Regarding the machine learning, the training-set AUC of 1.000 was likely inflated by small-sample overfitting and should be interpreted with caution. The limited sample size of the independent testing cohorts may have rendered per-cohort AUC estimates unstable, although the combined testing AUC of 0.798 was encouraging, it also warrants cautious interpretation. Validation in larger, multicenter cohorts is required to confirm the generalizability of the signature. ComBat correction cannot fully eliminate residual batch effects. The survival correlation analysis was based on a single retrospective TCGA cohort and remains exploratory, validation in independent prospective cohorts is needed. Finally, our model was built on baseline tumor transcriptomic data and therefore captures only the disease state at a single time point. Because acquired resistance develops over time, the model cannot predict when resistance will occur. Its clinical value lies in identifying high-risk patients at baseline, who may benefit from closer monitoring or alternative treatment strategies. Longitudinal studies with serial tumor biopsies or repeated plasma ctDNA testing are encouraged to understand how resistance evolves over time.

Above all, this study presents a potential 7 MORGs signature and suggests RRBP1 as a candidate regulator of OR. These findings warrant further investigation of RRBP1 as a potential candidate for overcoming EGFR-TKI resistance.

## Conclusion

In summary, we identified a 7-gene signature associated with OR in LUAD by integrating single-cell RNA-seq with bulk transcriptomic analyses. Our data revealed an increased proportion of M2 macrophages in OR and highlighted RRBP1 as the core candidate gene. *In vitro*, RRBP1 was upregulated in osimertinib-resistant H1975 cells, and its knockdown reduced cell viability, migration, and drug IC50, while increasing ER stress markers GRP78 and CHOP. These findings suggest that RRBP1 may contribute to osimertinib resistance, possibly through its effects on ER stress and macrophage-related microenvironment remodeling, although the underlying mechanisms warrant further investigation.

## Data Availability

The datasets analyzed for this study can be found in the GEO and TCGA. The bulk RNA-seq data are available under the accession numbers GSE193258, GSE146850, GSE278222, GSE276807 and GSE254485 (https://www.ncbi.nlm.nih.gov/geo/). TCGA data are available at the GDC Data Portal (https://portal.gdc.cancer.gov/projects/TCGA).

## Supplementary material

SUPPLEMENTARY FIGURE S1 Dot plot of marker genes for 22 cell clusters.

SUPPLEMENTARY FIGURE S2 PCA of uncorrected GEO Test cohort shows distinct dataset clustering, which is largely abrogated after correction.

## Glossary

AUC: Area Under the Curve
bulk RNA-seq: bulk RNA sequencing
CCK-8: Cell Counting Kit-8
CI: confidence interval
DEGs: differentially expressed genes
EGFR: epidermal growth factor receptor
ER: endoplasmic reticulum
FBS: fetal bovine serum
FPKM: fragments per kilobase of exon per million mapped reads
GAPDH: glyceraldehyde-3-phosphate dehydrogenase
GEO: Gene Expression Omnibus
GO-BP: Gene Ontology Biological Process
WGCNA: Weighted gene co-expression network analysis
HR: hazard ratio
IC50: half maximal inhibitory concentration
KEGG: Kyoto Encyclopedia of Genes and Genomes
LUAD: lung adenocarcinoma
ML: machine learning
MORGs: macrophage- and osimertinib resistance-related genes
NOR: non-osimertinib resistant
NSCLC: non-small cell lung cancer
OR: osimertinib resistance
PCA: principal component analysis
QC: quality control
RMA: robust multi-array average
ROC: receiver operating characteristic
RRBP1: ribosome-binding protein 1
RT-qPCR: quantitative real-time PCR
scRNA-seq: single-cell RNA sequencing
SD: standard deviation
SHAP: SHapley Additive exPlanations
siRNA: small interfering RNA
SNN: Shared Nearest Neighbor
STR: short tandem repeat
TAMs: tumor-associated macrophages
TCGA: The Cancer Genome Atlas
TKI: tyrosine kinase inhibitor
TME: tumor microenvironmen
TPM: transcripts per million
t-SNE: t-distributed Stochastic Neighbor Embedding
UMI: unique molecular identifier
UPR: Unfolded Protein Response
WGCNA: weighted gene co-expression network analysis
XGBoost: eXtreme Gradient Boosting
