# Supplementary material for: Integrative bioinformatics screening of a M2 macrophage-associated gene signature for osimertinib resistance
Source: Front Genet. 2026 Sep 28;17:1928986. doi: 10.3389/fgene.2026.1928986 (PMC13654588; doi:10.3389/fgene.2026.1928986)
Supplement: Supplementary file 1 [file DataSheet1.pdf]

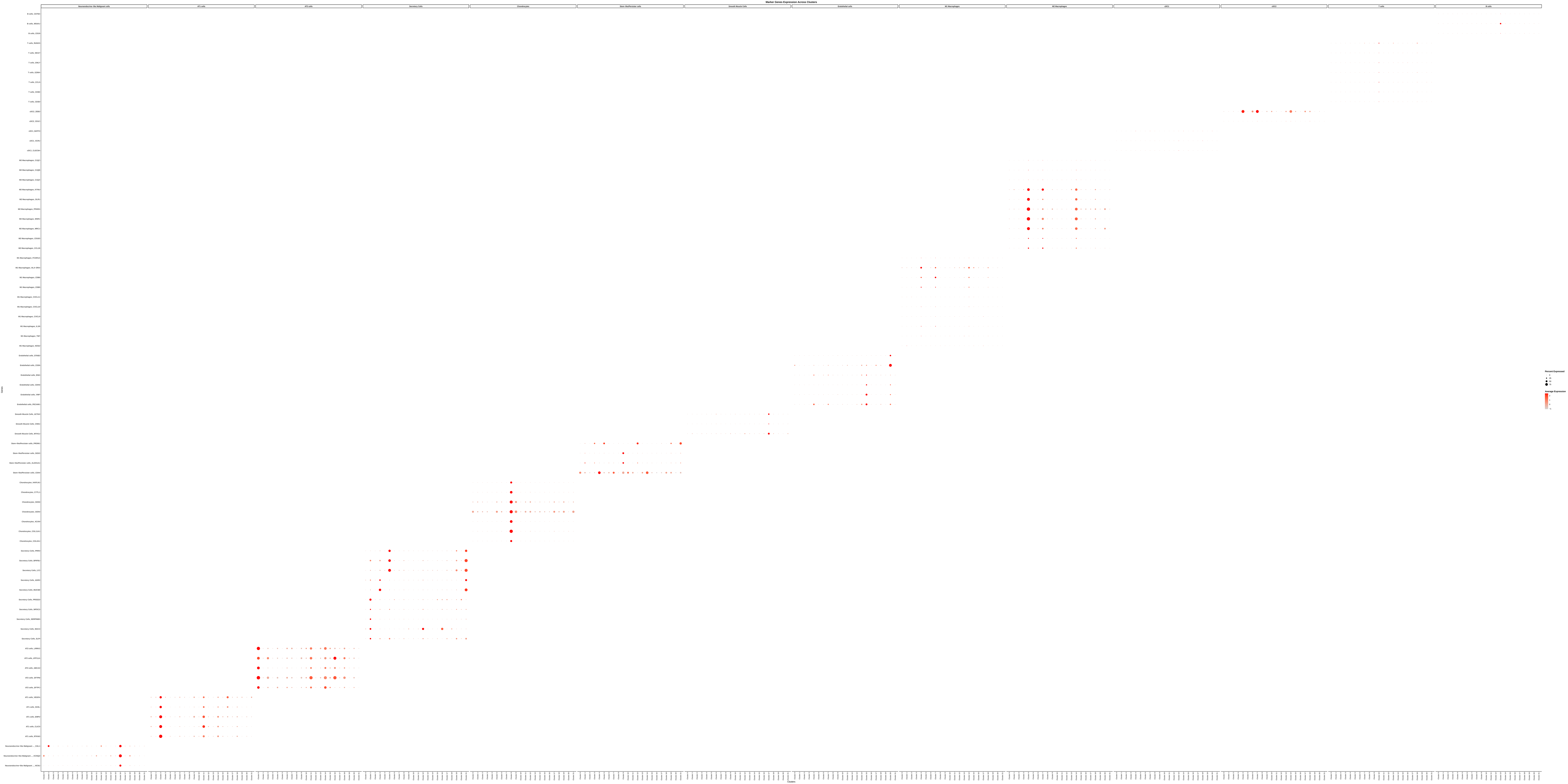
Marker Genes Expression Across Clusters
Genes
Clusters
Percent Expressed
Average Expression
