# Supplementary material for: Integrative bioinformatics screening of a M2 macrophage-associated gene signature for osimertinib resistance
Source: Front Genet. 2026 Sep 28;17:1928986. doi: 10.3389/fgene.2026.1928986 (PMC13654588; doi:10.3389/fgene.2026.1928986)
Supplement: Supplementary file 2 [file DataSheet2.pdf]

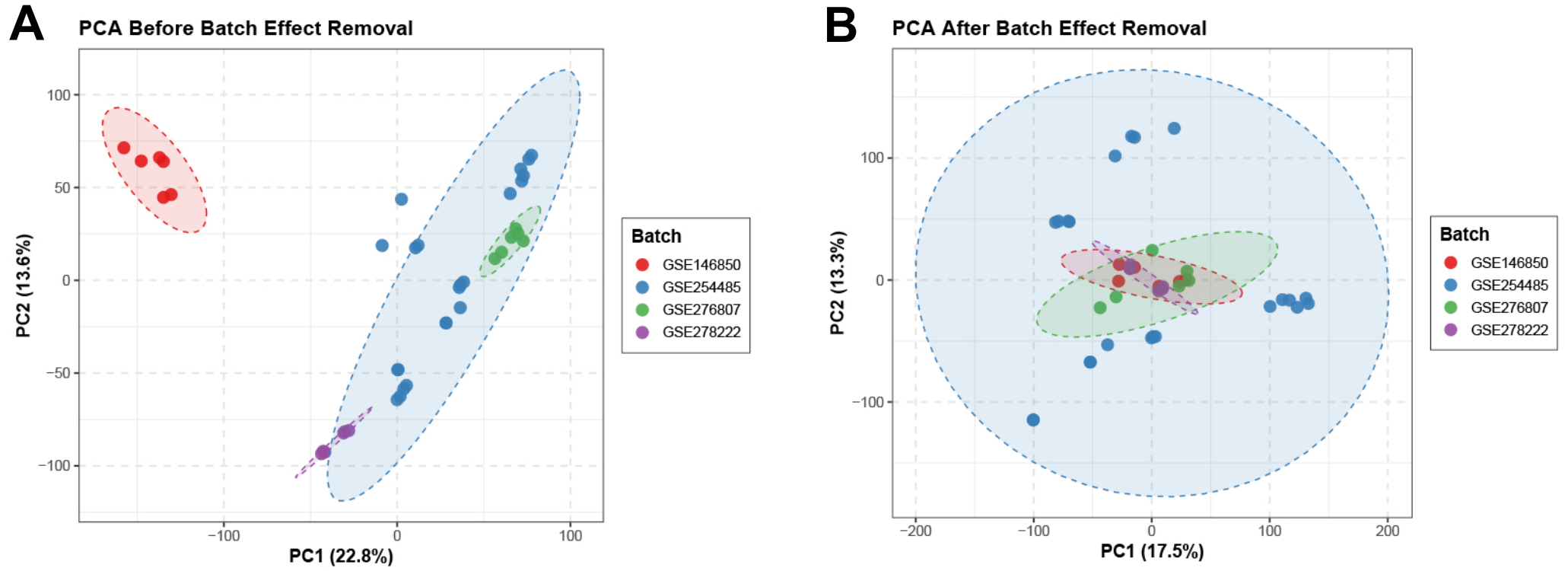
A
PCA Before Batch Effect Removal
100
50
0
−50
−100
−100
0
100
PC1 (22.8%)
PC2 (13.6%)
Batch
GSE146850
GSE254485
GSE276807
GSE278222
B
PCA After Batch Effect Removal
100
0
−100
−200
−100
0
100
200
PC1 (17.5%)
PC2 (13.3%)
Batch
GSE146850
GSE254485
GSE276807
GSE278222
