# Supplementary material for: Integrative bioinformatics screening of a M2 macrophage-associated gene signature for osimertinib resistance
Source: Front Genet. 2026 Sep 28;17:1928986. doi: 10.3389/fgene.2026.1928986 (PMC13654588; doi:10.3389/fgene.2026.1928986)
Supplement: Supplementary file 3 [file Table1.docx]

| Patient ID | Age | Gender | TNM stage | Clinical stage | Group | RECIST |
| --- | --- | --- | --- | --- | --- | --- |
| PA01 | 55 | female | T2aN1M0 | Ⅱb | NOR | CR |
| PA02 | 46 | male | T2bN1M0 | Ⅱb | NOR | CR |
| PA03 | 53 | male | T3N0M1 | ⅠV | NOR | PR |
| PA04 | 47 | male | T3N2bM1 | ⅠV | OR | PD |
| PA05 | 62 | male | T2bN0M1 | IV | OR | PD |
| PA06 | 54 | female | T1bNxM1c | Ⅳb | OR | PD |

Table SI. Clinicopathological characteristics of 6 LUAD patients enrolled in this study.
