# Supplementary material for: Integrative bioinformatics screening of a M2 macrophage-associated gene signature for osimertinib resistance
Source: Front Genet. 2026 Sep 28;17:1928986. doi: 10.3389/fgene.2026.1928986 (PMC13654588; doi:10.3389/fgene.2026.1928986)
Supplement: Supplementary file 4 [file Table2.docx]

| **Lot No** | **Oligo Name** | **Sequence(5' to 3')** | **Length** | **Purification** |
| --- | --- | --- | --- | --- |
| 1148993535 | RRBP1-1-s | gga uau uua cga cac uca a/dt//dt/ | 21 | RNase free HPLC |
| 1148993536 | RRBP1-1-a | uug agu guc gua aau auc c/dt//dt/ | 21 | RNase free HPLC |
| 1148993538 | RRBP1-2-s | gcu cug uag uga auu cca u/dt//dt/ | 21 | RNase free HPLC |
| 1148993539 | RRBP1-2-a | aug gaa uuc acu aca gag c/dt//dt/ | 21 | RNase free HPLC |
| 1148993541 | RRBP1-3-s | cag ugg aga aga aag gaa a/dt//dt/ | 21 | RNase free HPLC |
| 1148993542 | RRBP1-3-a | uuu ccu uuc uuc ucc acu g/dt//dt/ | 21 | RNase free HPLC |

Table SII. The sequences of si-RRBP1.
